# Characterizing the contribution of plasticity and genetic differentiation to community‐level trait responses to environmental change

**DOI:** 10.1002/ece3.3947

**Published:** 2018-03-23

**Authors:** Geneviève Lajoie, Mark Vellend

**Affiliations:** ^1^ Département des sciences biologiques Université du Québec à Montréal Montréal QC Canada; ^2^ Département de biologie Université de Sherbrooke Sherbrooke QC Canada

**Keywords:** climate change response, community trait variation, genetic differentiation, intraspecific trait variation, local adaptation, phenotypic plasticity, species turnover, transplant experiment

## Abstract

The match between functional trait variation in communities and environmental gradients is maintained by three processes: phenotypic plasticity and genetic differentiation (intraspecific processes), and species turnover (interspecific). Recently, evidence has emerged suggesting that intraspecific variation might have a potentially large role in driving functional community composition and response to environmental change. However, empirical evidence quantifying the respective importance of phenotypic plasticity and genetic differentiation relative to species turnover is still lacking. We performed a reciprocal transplant experiment using a common herbaceous plant species (*Oxalis montana*) among low‐, mid‐, and high‐elevation sites to first quantify the contributions of plasticity and genetic differentiation in driving intraspecific variation in three traits: height, specific leaf area, and leaf area. We next compared the contributions of these intraspecific drivers of community trait–environment matching to that of species turnover, which had been previously assessed along the same elevational gradient. Plasticity was the dominant driver of intraspecific trait variation across elevation in all traits, with only a small contribution of genetic differentiation among populations. Local adaptation was not detected to a major extent along the gradient. Fitness components were greatest in *O. montana* plants with trait values closest to the local community‐weighted means, thus supporting the common assumption that community‐weighted mean trait values represent selective optima. Our results suggest that community‐level trait responses to ongoing climate change should be mostly mediated by species turnover, even at the small spatial scale of our study, with an especially small contribution of evolutionary adaptation within species.

## 1 INTRODUCTION

Studies partitioning the relative contributions of intraspecific variation (ITV) and species turnover (SPT) to community‐level trait variation have provided novel insights into community assembly and responses to environmental gradients over space and time (Davis, Shaw, & Etterson, 2005; Cornwell & Ackerly, 2009; Lepš, de Bello, Šmilauer, & Doležal, 2011; Jung et al., 2014). A large importance of SPT suggests that the maintenance of trait–environment matching will depend on changes in species composition, which likely occur over relatively long timescales (Parmesan, 2006). Alternately, a large contribution of ITV suggests an ability of individual species to change their trait values to adapt to environmental change (Jump & Penuelas, 2005; Reed, Schindler, & Waples, 2011), which might occur relatively rapidly via phenotypic plasticity or more slowly via local adaptation. A few frameworks have been proposed to understand how the relative importance of ITV versus SPT varies across spatial scales (Albert, Grassein, Schurr, Vieilledent, & Violle, 2011) or how it depends upon the type of gradient and community under study (Lajoie & Vellend, 2015). Still, little attention in this literature has been paid to how the different sources of ITV—phenotypic plasticity versus genetic variation—might affect predictions of community functional response to environmental change.

Different sources of ITV have potentially important consequences for predicting the nature and pace of responses to environmental change. On one hand, trait variation mostly driven by phenotypic plasticity could mediate trait responses to environmental change very rapidly (e.g., within a single growing season). On the other hand, trait responses dominated by local adaptation necessitate microevolution over multiple generations, such that the maintenance of community trait–environment matching under this scenario would be driven by the slower process of turnover in the genetic composition of populations. Species distribution models incorporating plasticity indeed predict reduced loss of distribution area under climate change compared with models based on presence–absence data only (Nicotra et al., 2010; Garzon, Alia, Robson, & Zavala, 2011; Valladares et al., 2014). In models accounting for local adaptation, the maintenance of species distribution area mostly depends on the potential for dispersal of the best‐adapted populations (Garzon et al., 2011; Valladares et al., 2014).

There is a long history of transplant experiments aimed at partitioning the environmental (plastic) and genetic sources of intraspecific variation in natural settings (Clausen, Keck, & Hiesey, 1940; Chapin & Chapin, 1981; Angert & Schemske, 2005). In such experiments, trait variation explained by the site of planting (where plants of a given origin are grown) is attributed to plasticity, while trait variation explained by the site of origin (where a plant at a given site came from) is attributed to genetic differentiation. However, few field‐based studies explicitly report the relative effect size of each process in driving ITV in a single experiment (but see Richardson, Chaney, Shaw, & Still, 2017), with meta‐analyses generally reporting vote counts of how many studies provide evidence for each source of ITV (Franks, Weber, & Aitken, 2014) rather than quantitative estimates of their relative importance along gradients. Reciprocal transplant studies do not typically include data on interspecific trait variation as a frame of reference when assessing sources of ITV, which further prevents generalizations regarding the importance of plasticity and genetic variation in maintaining community‐level trait–environment matching.

Different studies have made strongly contrasting assumptions about the importance of plastic versus genetic responses to environmental change. For example, Siefert et al. (2015) proposed that the low heritability generally observed in plant functional traits (Geber & Griffen, 2003) suggests a dominance of plastic relative to genetic variation in most communities. Chevin, Collins, and Lefèvre (2013) further suggest that plasticity may be the only effective response to rapid climate change, given that genetic changes occur too slowly. Other studies however tend to emphasize a major role of local adaptation and the potential for microevolutionary changes in mediating trait–environment matching among communities (Leimu & Fischer, 2008; Laughlin & Messier, 2015).

Our overarching objective in this study was to characterize the contributions of plasticity versus genetic variation to trait variation along an elevational gradient where we previously quantified the contribution of species turnover at the community level (Lajoie & Vellend, 2015). To do so, we conducted a reciprocal transplant experiment of an herbaceous plant species, *Oxalis montana* Raf., across a mountainside in southern Québec, Canada. We focused on a relatively short spatial gradient (~300 m elevation change) that represents a realistic magnitude of temperature change (~2°C) expected over the next 50–100 years (Ouranos 2015) and that spans the two major forest biomes (temperate and boreal) in eastern North America. We provide one of the first interpretations of the nature and strength of the intraspecific functional response in the context of community trait turnover studied previously (Lajoie & Vellend, 2015), although we recognize that quantifying the components of ITV with one experimental species represents just a first step in this line of research. In the earlier study, we partitioned turnover in community‐weighted mean traits of herbaceous communities into their intraspecific and interspecific components for three major plant functional traits. In this study, we were further interested in testing the common assumption of trait‐gradient studies that local community‐level trait means (weighted by species abundances) represent adaptive optima (Shipley, de Bello, Cornelissen, Laliberté, & Reich, 2016). If this assumption is valid, we expect that plants with trait values closest to the community mean should display the highest fitness. In addition, if community‐level trait–environment correlations represent adaptive responses to the gradient in question, then traits with strong environmental correlations should also be good predictors of individual fitness variation within species across the gradient. Here, we address the following specific questions:

1. What is the relative importance of plasticity versus genetic differentiation in driving variation in functional traits and fitness components among populations of *O. montana*?
2. Is there evidence for local adaptation?
3. How does the magnitude of plastic and genetic contributions to intraspecific variation compare with species turnover in explaining community‐level trait change along the elevational gradient?
4. Do community‐based gradient analyses predict the direction of selection within sites and the traits most strongly related to fitness?

## 2 MATERIALS AND METHODS

### 2.1 Study site

In 2012, we established a transplant experiment along an elevational gradient spanning a transition from deciduous forest (low elevation) to coniferous forest (high elevation), in Mont‐Mégantic National Park (45°27′21″N, 71°09′08″W), southern Quebec, Canada. Over the 300‐m elevation span of our study plots, we have observed a decline in mean annual temperature of 1.9°C (2.3 at ~700 m a.s.l. to 0.4°C at ~1,000 m a.s.l.), and a strong gradient in community structure and functional composition (Lajoie & Vellend, 2015). Further information on the study site, along with a description of understory plant species and trait turnover along this gradient, may be found in earlier studies (Brown & Vellend, 2014; Savage & Vellend, 2015; Lajoie & Vellend, 2015).

### 2.2 Study organism

The relative importance of phenotypic plasticity and genetic differentiation in creating a match between traits and environment was assessed for *Oxalis montana* Raf. (Oxalidaceae). This species is a small perennial herbaceous plant spanning the entire elevational gradient at our study site, being more abundant in the cool and shaded understory of boreal forests than in deciduous forests. *Oxalis montana* may reproduce by outcrossed seed and facultatively by self‐fertilizing cleistogamous (non‐opening) flowers (Berg, 2000). It also propagates vegetatively by rhizomes which degrade within 1 or 2 years upon establishment of the new individual, making it difficult to retrace the genetic origin of individuals based on rhizome networks. It was chosen as one of the few species that was common across the whole gradient and representative of the perennial lifestyle of most understory species in this forest.

### 2.3 Transplant experiment

**Figure 1:**
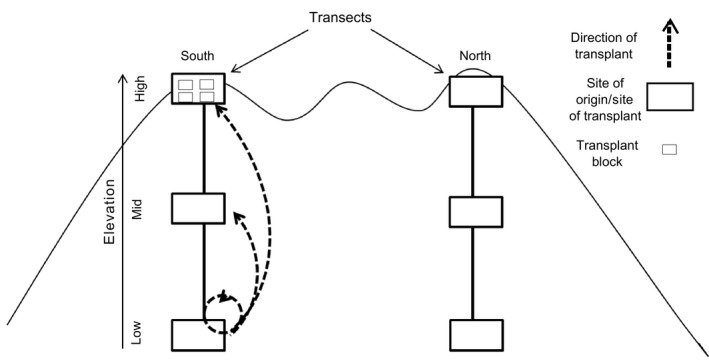
Reciprocal transplant experiment design. Individuals from each site of origin were transplanted to all three elevations (low, mid, and high) within the same transect of origin (long‐dashed arrows). At each transplant site, individuals were randomly separated among four blocks, by site of origin. Transplant sites at low and high elevations were separated by approximately the same distance as the two transects (~1 km). Two datasets were defined, consisting of intra‐ and intertransect transplants. Direction of transplants is indicated for only one of the sites (south transect, low elevation), as well as the transplant blocks (south transect, high elevation)

Our experimental system consisted of six sites, distributed among two transects and three elevations: low (~715 m a.s.l.), medium (~870 m a.s.l.), and high (~1,040 m a.s.l.) (Figure 1). In spring 2012 (30 May to 12 June), 140 individuals were randomly selected at each site and transplanted to a common garden at ~400 m elevation, with the objective of minimizing maternal effects prior to planting back into the field the next season. The size of each individual was first standardized approximately by cutting rhizomes to a maximum length of 50 mm (min = 6 mm). Individuals were then planted in pairs in pots filled with a soil mixture composed of 10:10:1 parts organic blend topsoil, sphagnum moss, and sand, with cedar mulch added on the surface after planting. Pots were placed under 50% shade cloth on an open lawn from June to October 2012, where pots were watered, weeded, and cleared of pests (slugs) as needed. In autumn 2012 (6–19 October), prior to the first frost, 84 individuals were selected per site of origin among those that survived, divided randomly into three groups (one per transplant site) and transplanted back in the field following the experimental design presented in Figure 1.

At each transplant site, we established four blocks in microhabitats where *O. montana* would normally grow, especially in mossy and humid patches at the shaded bases of trees. The 28 individuals of each site of origin attributed to a transplant site were randomly attributed to each of these four blocks, such that each block contained seven individuals from each origin. Previous experiments with *O. montana* have reported strong plastic responses of this species to its light environment (Packham & Willis, 1977), so we avoided prominent canopy gaps (rare at all sites) when selecting blocks. Low‐elevation forest understories experience high light in early spring and late fall (before tree leaf‐out and after leaf‐fall, respectively), although we consider this as part of a long‐term response to the climatic gradient (deciduous trees in warmer sites, conifers in cooler sites) rather than as a factor confounding our interest in the climatic component of the elevational gradient. In a 40‐cm diameter circle around each individual, we carefully cleared other *O. montana* individuals (but not other species), in order to ensure our ability to follow each individual's subsequent clonal growth. Plants were left in the field for 1 year, after which time surviving individuals were harvested and brought back to the laboratory for measurements (6–14 October 2013).

### 2.4 Fitness components

Individuals that died during the winter were excluded from the study, their death ascribed to transplant shock. Two fitness components were scored for the remaining individuals during the experiment. First, summer survival was assessed at the end of the growing season 2013. Plants were considered to have survived either with or without leaves, as long as the rhizome was still alive. Living rhizomes were recognized by their bright white color and turgidity. Second, total dry biomass (above‐ and below‐ground) at the end of the growing season 2013 was taken as a component of fitness, allowing comparisons among surviving individuals. Each harvested specimen was oven‐dried at 70°C for 72 hr, after which dry weight was measured. Dry weight of the leaf sampled earlier for measuring SLA (see below) was added to the individual's biomass.

### 2.5 Trait measurements

We aimed to measure the same three functional traits that were investigated in our community‐level study (maximum height, specific leaf area (SLA), and reproductive phenology), with the addition of leaf area, as these traits represent major axes of adaptive plant strategies with known associations with plant fitness. However, few *O. montana* plants flowered (even for mature plants in the field, many do not flower in a given year at our study site), so our analyses did not include reproductive phenology. Maximum foliage height, indicative of a plant's ability to access light (Givnish, 1982), was measured with a ruler to the nearest millimeter on plants in the field in midsummer (26–30 July). SLA, a correlate of photosynthetic rate reflecting a trade‐off between resource capture and conservation (Reich, Walters, Ellsworth, & Uhl, 1994), was assessed by taking the second largest leaf (without petiole) from all plants possessing at least two healthy leaves toward the end of the growing season but before leaf senescence (20–26 August). Leaves were first scanned, then dried at 70°C for 72 hr, and weighed. Leaf area, itself a trait reflecting water use and light capture strategies, was measured from scanned leaves using ImageJ (Rasband, 1997–2012). SLA was then calculated as leaf area/dry weight.

### 2.6 Partitioning genetic and environmental sources of intraspecific variation

Our experimental design allowed us to partition the genetic (G) and environmental/plastic (E) contributions to intraspecific variation observed across elevations and transects (using type III ANOVAs, as described below). For assessing elevation effects on each trait and fitness component, we built a model predicting trait variation as a function of the elevation of origin (EL_O_), the elevation of transplant (EL_T_), and their interaction. Transect (*R*) was included in the model as a fixed factor given that we had only two transects. Block, nested within elevation of transplant and transect, was included as a random factor. Initial biomass (Init.biom) was also added as a covariate to each model (calculation described below). We ran the full model (Equation (1)) using the R package lme4 (Bates, Maechler, Bolker, & Walker, 2015).

(1) $$\text{Trait}\;∼\;{\text{EL}}_{O}\;+\;{\text{EL}}_{T}\;+\;{\text{EL}}_{O}:{\text{EL}}_{T}\;+\;R\;+\;\left(1|R:{\text{EL}}_{T}:\text{Block}\right)\;+\;\text{Init.biom}$$

where the notation “1|” introduces our random factor and the sign “:” indicates nesting of right‐hand terms into left‐hand ones.

Because we could not measure dry biomass directly prior to transplanting, we predicted initial dry biomass (Init.biom) from morphological characteristics measured prior to transplant, using a stepwise regression model explaining final dry biomass as a function of these same morphological characteristics measured at the end of the experiment. The final model included four explanatory variables (height, rhizome length, rhizome thickness, and number of leaves) and explained 74% of variation in dry biomass (details in Appendix 1). For survival data, a binary response, a general linear model with a binary family function was used, while linear models were used for continuous quantitative traits. In every case, trait data were transformed to respect assumptions of normality: A square‐root transformation was used for height while SLA and leaf area were both log‐transformed.

A type III ANOVA, appropriate for unbalanced designs, was then used to partition variation due to phenotypic plasticity (*E*, variation explained by the elevation where a plant was grown) or genetic differentiation (*G*, variation explained by the elevation of origin). An effect of the interaction term (*E* × *G*) when individuals have higher fitness than foreigners in their site of origin can be interpreted as local adaptation (Kawecki & Ebert, 2004). In order to test for differences between origins in each elevation of transplant, we performed post hoc pairwise comparisons on the models explaining survival and final dry biomass using the R package lsmeans (Lenth, 2016) (Appendix 2).

The relative importance of phenotypic plasticity (%*E*) in explaining variation in a given trait was calculated as the ratio between the sum of squares explained by *E* (SS_*E*_), and the sum of the sum of squares explained independently by *E* (SS_*E*_) and *G* (SS_*G*_) in the ANOVA (Equation (2)).

(2) $$\%E\;=\;\frac{{\text{SS}}_{E}}{{\text{SS}}_{E}\;+\;{\text{SS}}_{G}}$$

The *G* × *E* term was not included in these calculations as the variation cannot be assigned clearly to either *G* or *E*.

### 2.7 Community analyses

Community‐level analyses performed previously (Lajoie & Vellend, 2015) consisted of partitioning trait variation among communities into their intraspecific (ITV) and interspecific (SPT) components. Here, we describe our general approach and refer readers to the earlier paper for details (see also Lepš et al., 2011). In a dataset with a total of 51 species, we first calculated community‐weighted mean traits in 30 sites across three transects (10 elevations per transect) in two ways: (1) using plot‐based trait values (including ITV) and (2) using species mean trait values (excluding ITV). For each of (1) and (2), we then calculated the variance in community mean traits that could be explained by elevation. The difference between the two is then attributable to ITV alone. We then quantified the relative contribution of ITV versus SPT in driving community trait turnover by calculating a ratio between the explanatory power of ITV and that of the two processes together. Our initial analyses were performed for three traits (height, SLA, and peak flowering date), to which we here add an equivalent and new analysis for leaf area.

### 2.8 Adaptive significance of traits

In order to test whether trait–environment matching at the community level represents adaptive responses to the gradient, we assessed whether selection in *O. montana* populations across elevations was in the direction of community‐weighted trait means measured along the same gradient. Natural selection at each site was assessed for all three traits using both standardized selection differentials (*S*) and linear selection gradients (β) (Lande & Arnold, 1983). The former (*S*) is calculated as the covariance between relative fitness (fitness/average fitness across individuals—using final biomass as a proxy for fitness in the formula) and a given standardized trait. Significance is then assessed using the *p*‐values of the Pearson correlation between the two variables. The latter (β) are calculated as the partial coefficients of multiple regression models of relative fitness versus all traits, thereby providing an estimate of the importance of each trait, controlling for all other traits in the model. The direction of these gradients was then compared with community‐weighted means (CWM) calculated as the sum of the products of the local trait values of each species of a community with their relative abundances in that community (Shipley, Vile, & Garnier, 2006). The prediction is that selection is in the direction of the CWM. All analyses were performed in R version 3.2.2 (R Core Team 2015).

## 3 RESULTS

### 3.1 Environmental versus genetic effects in driving intraspecific trait and fitness variation

**Figure 2:**
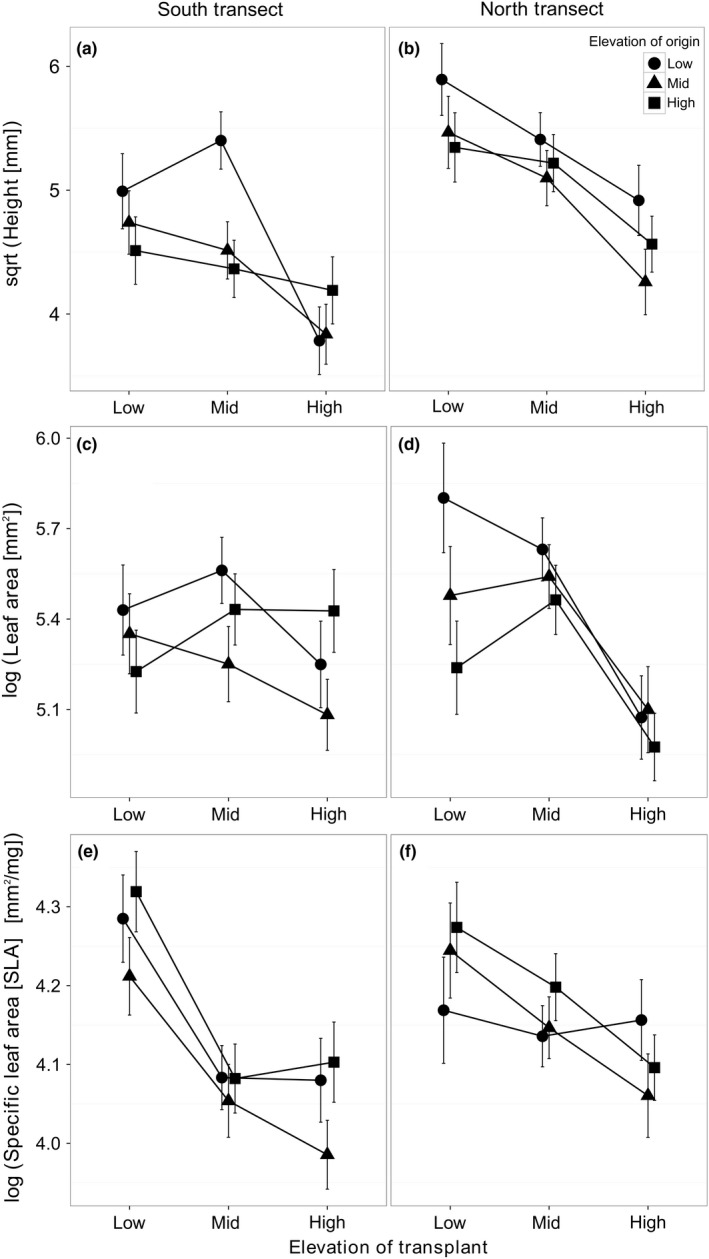
Variation in three traits (a, b: height; c, d: leaf area; and e, f: specific leaf area) among *Oxalis montana* populations originating from three different elevations (low, mid, and high) and transplanted to each of these elevations, within each of two transects (columns). Dots represent least squares means of transformed population mean trait values, accounting for initial biomass, with symbols identifying elevations of origin. Standard errors are presented for each mean

Elevation of transplant (EL_T_) was the strongest driver of trait differences among *O. montana* populations for the three traits measured (height, leaf area, and SLA), all tending to decrease from low to high elevations (Table 1A, Figure 2). Elevation of origin (EL_O_) explained a significant portion of trait variation only for height (Table 1A). We found no evidence for differences in plasticity among population origins (i.e., no significant EL_O_ × EL_T_ effects, Table 1A).

**Table 1:** Analyses of variance on traits (A) and fitness components (B) measured on experimental *Oxalis montana* individuals from three different elevations of origin transplanted to each of three elevations, within two different transects. The table shows degrees of freedom (*df*), mean squares (MS), and *F*‐ratios for quantitative continuous variables (all traits and biomass) and degrees of freedom and chi‐square statistics (Chisq) for the binary variable (survival). Degrees of freedom for continuous variables were approximated with the Satterthwaite method implemented in R package lmerTest. Significance is indicated as the following: *p < .05, **p < .01, ***p < 0.001

(A) Traits									
	Height			Leaf area			Specific leaf area (SLA)		
	*df*	MS	*F*	*df*	MS	*F*	*df*	MS	*F*
Elevation of origin (EL_O_)	2	4.75	4.43*	2	0.711	2.74	2	0.099	2.90
Elevation of transplant (EL_T_)	2	10.26	9.57**	2	2.224	8.58**	2	0.355	10.38***
EL_O_ X EL_T_	4	1.12	1.04	4	0.265	1.02	4	0.028	0.83
Transect (R)	1	13.50	12.58**	1	0.038	0.15	1	0.044	1.28
Biomass covariate	1	0.15	0.14	1	0.043	0.17	1	0.189	5.53
Residuals	315	1.10		271	0.267		271	0.035	

(B) Fitness components					
	Survival		Biomass		
	*df*	Chisq	*df*	MS	*F*
Elevation of origin (EL_O_)	2	1.04	2	0.011	2.28
Elevation of transplant (EL_T_)	2	5.61	2	0.008	1.61
EL_O_ × EL_T_	4	3.24	4	0.015	3.10*
Transect (R)	1	0.00	1	0.005	1.04
Biomass covariate	1	0.14	1	0.018	3.78
Residuals	374		298	0.005	

**Figure 3:**
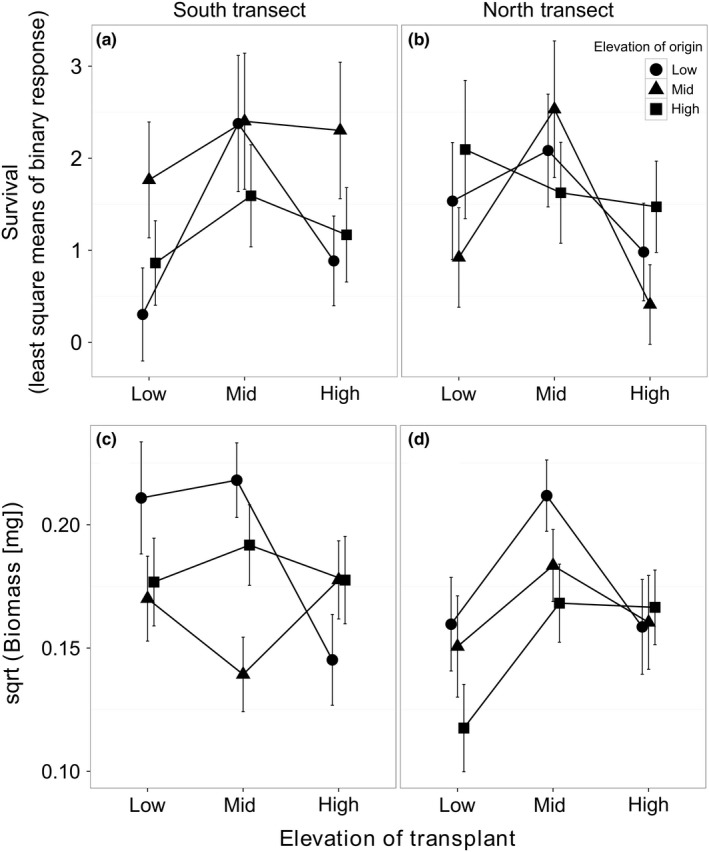
Variation in survival (a,b) and biomass (c,d) among *Oxalis montana* populations originating from three different elevations (low, mid, and high) along a mountainside and transplanted to each of these elevations, within each of two transects (columns). Dots represent least squares means of transformed population mean values, accounting for initial biomass, with symbols identifying elevations of origin. Standard errors are presented for each mean

Variation in survival and biomass across our gradient were explained only to a small extent by either elevation of transplant, elevation of origin, or their interaction (Figure 3, Table 1B). Survival was highest at midelevation sites for all origins except the high‐elevation individuals from the north transect (Figure 3a,b). For biomass, only the EL_O_ × EL_T_ interaction was significant (Table 1B), but pairwise comparisons show that this effect was driven by only one significant pairwise difference, observed between populations of low‐ and midelevation origins transplanted at midelevation (see Section 2.1 in Appendix 2).

**Figure 4:**
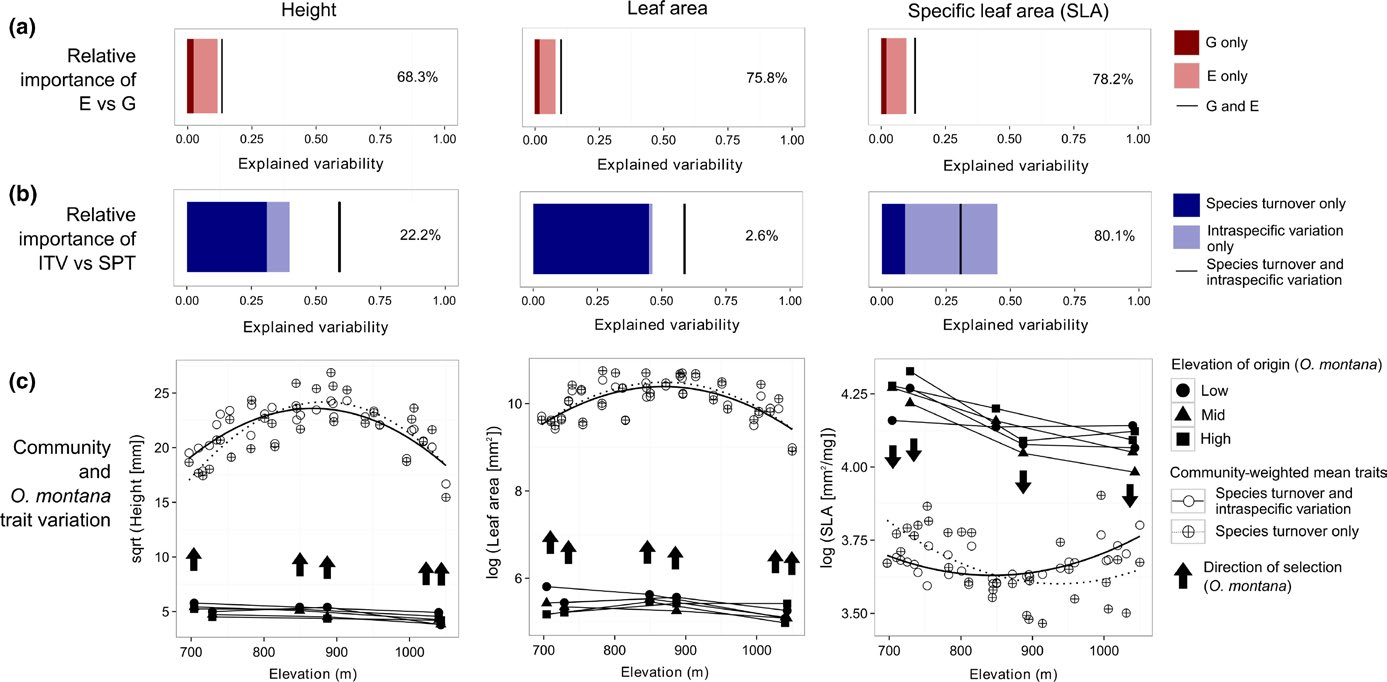
Community‐level and *Oxalis montana* trait variation across the elevational gradient and their variance partitioning. (a) Variation in *O. montana* traits that can be explained respectively by genetic (dark red bar) and environmental (pale red bar) effects, relative to the total variation explained by either (black line). The importance of E relative to G is indicated to the right of the stacked bars for each trait. (b) Community‐level trait turnover (data from Lajoie & Vellend, 2015) that can be explained respectively by species turnover (SPT: dark blue bar) and intraspecific variation (ITV: pale blue bar) relative to the total variation explained by both (black line). The importance of ITV relative to SPT is indicated to the right of the stacked bars for each trait. In both a and b, the space between the end of the bar and the black line represents covariation between the two components of trait change (positive if the black line is further to the right than the stacked bars, and vice versa). (c) Community‐weighted trait means (open symbols, data from Lajoie & Vellend, 2015) and *O. montana* population trait means (black symbols, as calculated for Figure 2) measured at each transplant site along the elevational gradient. Community‐weighted means were calculated using species population trait means measured at each site (including species turnover and intraspecific variation effects), or average species trait means across the gradient (including only species turnover effects). Arrows represent the direction of standardized selection differentials calculated for each transplant site as presented in Table 2. Only significant selection differentials are shown

The relative importance of phenotypic plasticity (E) was large and consistent among traits, varying between 68.3% and 78.2% (Figure 4a). These uniformly large proportions contrast with the highly variable proportional contributions of ITV (relative to SPT) in explaining community‐level trait–elevation relationships, which ranged from 2.6% to 80.1% for these three traits (Figure 4b). Despite the fact that elevation was a significant driver of trait variation in *O. montana*, overall, it still accounted for a low proportion (~13%) of the total variance in intraspecific trait values observed among individuals, most variance remaining unexplained in the model (Figure 4a).

### 3.2 Is trait variation adaptive?

The strength of selection on traits varied across the gradient, but the direction of selection was generally consistent across elevations (Table 2). For all three traits, individuals with traits closest to the community‐weighted mean trait had higher biomass (Figure 4c, Table 2). Plants with lower SLA had greater fitness in *O. montana* populations at all three elevations. Fitness also increased with increasing height and leaf area. While selection differentials (*S*) indicate many significant associations between dry biomass and variation of all traits (Table 2), the larger selection gradients (β) and selection differentials of leaf area relative to height and SLA at all sites point to the predominant importance of this trait, or covariation of this trait with others, in explaining these relationships in a consistent way across elevations.

**Table 2:** Site‐specific analyses of selection. Standardized selection differentials (*S*) were calculated as the covariance between relative fitness and measured traits, and standardized directional selection gradients (β) were measured from multiple regression linear models predicting relative fitness as a function of three traits, in each of the six transplant sites (low, mid, and high‐elevation sites over two transects). Standard errors are indicated in parentheses. Adjusted *R* ^2^ of the selection gradient models are indicated at the bottom of the table for each site. Significance is indicated as the following: *p < .05, **p < .01, ***p < .001

		South transect			North transect		
		Low	Med	High	Low	Med	High
Height	*S*	0.181^†^	0.172*	0.139*	0.502*	0.284***	0.282**
	β	−0.156 (0.084)^†^	−0.017 (0.090)	0.025 (0.098)	0.078(0.202)	0.123 (0.096)	0.135 (0.117)
Leaf area	*S*	0.441***	0.314***	0.307***	0.633**	0.489***	0.478***
	β	0.516 (0.084)***	0.490 (0.111)***	0.423 (0.087) ***	0.452 (0.231)^†^	0.237 (0.069)**	0.893 (0.152)***
Specific leaf area (SLA)	*S*	−0.209*	−0.193**	−0.136^†^	−0.332*	−0.114	−0.356**
	β	−0.085 (0.068)	−0.236 (0.095)*	−0.178 (0.078)*	−0.196 (0.246)	−0.092 (0.080)	−0.122 (0.102)
Adj. *R* ^2^ (β models)		.64***	.38***	.48***	.26	.32***	.67***

## 4 DISCUSSION

Most studies aiming to quantify the relative importance of ecological and evolutionary processes in driving community trait turnover have so far focused on (1) partitioning interspecific and intraspecific sources of variation without reference to underlying causes of ITV (references within Siefert et al., 2015), or (2) individual species and the contribution of plasticity or genetic differentiation to trait variation, without reference to the community context (e.g., Etterson & Shaw, 2001; Gonzalo‐Turpin & Hazard, 2009). Our study explicitly quantified the relative role of each of these sources of community‐level trait variation in the same system, along an elevational gradient. Although we partitioned sources of intraspecific variation in only one species, we believe our results remain relevant at the community‐level, given that experiments in which parallel transplants were performed with several species have found similar drivers of intraspecific variation across species (Angert & Schemske, 2005; Poll, Naylor, Alexander, Edwards, & Dietz, 2009; Frei, Ghazoul, Matter, Heggli, & Pluess, 2014). We further make use of selection gradients calculated from individual‐level data to address the rarely challenged assumption that community trait–environment matching represents adaptive responses to a gradient (Muscarella & Uriarte, 2016; Shipley et al., 2016).

### 4.1 A dominant role of plasticity in driving intraspecific trait variation

Overall, elevation of transplant was the most important factor explaining trait variation among populations of *O. montana*, indicating a major effect of phenotypic plasticity underlying intraspecific trait variation. Genetic differentiation among populations (effects of elevation of origin) made a comparatively very small contribution. Our results support evidence from other transplant experiments of herbaceous plants, in which plasticity has been documented as a predominant driver of intraspecific variation in SLA (Meziane & Shipley, 1999; Gonzalo‐Turpin & Hazard, 2009; Mitchell & Bakker, 2014; but see Etterson, 2004) and plant height (Chapin & Chapin, 1981; Emery, Chinnappa, & Chmielewski, 1994; Gonzalo‐Turpin & Hazard, 2009). Roughly equal contributions of plastic and genetic effects have however been observed in the literature for leaf size (Chapin & Chapin, 1981; Emery et al., 1994; Hautier, Randin, Stocklin, & Guisan, 2009).

Several ecological factors could increase the relative importance of plastic versus genetic responses to the gradient investigated. First, despite potential for divergent selection due to marked environmental change with elevation (Lajoie & Vellend, 2015), and short seed dispersal distances in our study species (reaching 1–2 m—Berg, 2000), the relatively small spatial scale under study (325 m of elevation, or ~1 km on the ground between low and high‐elevation populations) suggests ample opportunity for gene flow among populations, which could reduce genetic differentiation. We have indeed observed overlap of *O. montana* flowering schedules between low‐ and high‐elevation sites despite differences in peak flowering dates (G. Lajoie & M. Vellend, unpublished data). Second, the perennial life habit of many species of the forest understory like *O. montana* is expected to favor adaptive phenotypic plasticity, as perennial individuals must be able to persist under substantial environmental variation within and between years (Van Tienderen & Van Der Toorn, 1991; Price & Marshall, 1999), especially in a temperate climate with dramatic intra‐annual climatic variation.

### 4.2 Weak evidence of local adaptation

Plasticity was an important driver of variation for functional traits but less so for fitness components. We did not find clear evidence of local adaptation sensu Kawecki and Ebert (2004) in our study system either (Table 1, Appendix 2). The elevation of origin × elevation of transect (EL_O_ × EL_T_) effect in our model for biomass was significant, but post hoc pairwise comparisons revealed only one significant difference between origins, not itself indicative of local adaptation (Figure 3, Appendix 2). The short duration of our experiment, representing a small portion of the life of a perennial plant, could have limited our capacity to detect genetically based differences in fitness. As biomass represents an imperfect proxy of fitness, a life‐time assessment of offspring production, although impractical to measure in long‐lived perennials, would permit more robust evolutionary inferences.

### 4.3 Inferring species response to environmental change

We provided an explicit measure of the relative importance of plasticity and genetic differentiation among populations in driving intraspecific variation along a climatic gradient, showing that the response to elevation was dominated by plasticity, despite evidence from the literature supporting a role for both drivers of ITV (Gienapp, Teplitsky, Alho, Mills, & Merilä, 2008; Leimu & Fischer, 2008; Reed et al., 2011; Franks et al., 2014). Microevolution is therefore unlikely to contribute in a major way to *O. montana*'s trait responses to environmental change, despite suggestive evidence to the contrary from a few studies (Franks, Sim, & Weis, 2007). If one was to predict the response of this species to climate change based on this transplant experiment (Davis et al., 2005), we would expect the predominant response to involve rapid plastic change, at least for climate changes of the magnitude observed along our gradient (~2°C). It is of course possible that a greater magnitude of warming, or changes in other drivers of global change (e.g., canopy opening via insect outbreaks), could involve more pronounced or different evolutionary responses.

Strongly contrasting viewpoints have emerged regarding the relative importance of plasticity and genetic variation in mediating species responses to environmental change. Citing a review by Geber and Griffen (2003), Siefert et al. (2015) submits that plasticity should be of greater magnitude than local adaptation “in most traits and communities” because of the low heritability of many plant functional traits in nature. Chevin et al. (2013) add that the slow rate at which natural selection proceeds may be insufficient to allow adaptive evolution to have a major influence on responses to rapid environmental change, such that plastic responses should be more important. Other studies emphasize the importance of local adaptation, notably by assuming phenotypic trait covariance matrices are determined mostly by genes rather than the environment (Laughlin & Messier, 2015). Our results clearly support the view that plasticity contributes more than local adaptation to spatial trait variation.

The relative importance of G and E in maintaining trait–environment relationships could however differ across different kinds of gradients (i.e., other than elevation). In our previous study, ITV made larger contributions to community‐level trait variation along gradients of nonclimatic factors such as light and soil pH than it did to the elevational gradient (Lajoie & Vellend, 2015). A different transplant study with *O. montana* also reported strong plastic responses to variable light environments (Packham & Willis, 1977). We found that relatively little trait variation in *O. montana* was explained by plastic or genetic responses to the elevational gradient, suggesting the possibility that trait variation is more strongly influenced by unmeasured environmental factors that vary within a given elevational zone, such as light or soil characteristics.

To the extent that our study species is representative of other perennials in our system, even the dominant component of ITV (phenotypic plasticity) would, however, be of only minor importance compared with species turnover in maintaining the fit between community‐level traits and a changing environment (Figure 4). We would predict the contribution of species turnover to remain high in the future, especially as plasticity reaches its limits under sustained environmental change. Future studies examining the relative importance of environmental versus genetic sources of ITV and also their magnitude relative to species turnover across several environmental axes and for several co‐occurring species will constitute a valuable area of investigation, in particular to improve predictions of community response to nonclimatic environmental change (see Brown & Vellend, 2014).

### 4.4 The adaptive significance of community‐level trait means

Studies of community‐level traits along environmental gradients often assume that the correspondence between average trait values and environmental variables is indicative of the adaptive nature of trait variation along that gradient (Ackerly, 2003). In order to build a strong case for predicting community responses to global change, one however must understand the adaptive link between traits and the environment (see Laughlin & Messier, 2015). We here found that in accordance with the CWM‐optimality hypothesis, the individuals displaying the trait values closest to the community‐weighted means consistently displayed higher fitness (Figure 4c), thereby suggesting these aggregated trait measures used in trait‐gradient analysis studies are useful predictors of the direction of selection within sites. In the case of plant height, one must interpret the selection analyses with some caution given that height and biomass are to some extent related allometrically (see Appendix 1). Our individual‐based results are coherent with a recent study reporting a negative correlation between species local abundance and the distance of their population mean trait values to the community‐weighted mean (Muscarella & Uriarte, 2016). Further tests of these relationships considering more traits and more species would contribute to the validation of this assumption.

In sum, we have conducted one of the first studies quantifying the importance of all three components underlying community‐level trait–environment relationships: plasticity, genetic variation, and species turnover. Our transplant experiment provided evidence for a greater role of phenotypic plasticity than local adaptation in driving intraspecific trait variation along the elevational gradient, suggesting the potential for rapid plant responses to short‐term environmental change. Our combination of population‐ and community‐level data, however, indicates that in this system, species turnover is likely to dominate community‐level responses to environmental change on the longer term, relegating plasticity and especially adaptive evolution to comparatively minor roles. Our results were further consistent with the frequent assumption that community‐weighted means represent trait optima, at least considering the three traits examined in our study species. Overall, our study contributes to refining predictions of the speed and nature of community response to environmental change while highlighting the insights gained from population‐level studies into community ecology.

## CONFLICT OF INTEREST

None declared.

## AUTHOR CONTRIBUTIONS

GL and MV conceived the ideas and designed methodology; GL collected and analyzed the data; GL led the writing of the manuscript. Both authors contributed critically to the drafts and gave final approval for publication.

## DATA ACCESSIBILITY

Data available from the Dryad Digital Repository: https://doi.org/10.5061/dryad.62g52g4 .

## APPENDIX 1

### Prediction of initial plant dry biomass

We built a multiple linear regression model to predict plant dry biomass from morphological measurements. We first built a model that aimed to explain variation in final dry biomass as a function of six explanatory variables measured at the end of the experiment: height, rhizome length, number of clonal shoots, maximal rhizome node thickness, maximal rhizome internode thickness, and number of leaves. Performing stepwise regression on this model, we obtained a final model explaining 74% of variation, which we then used to predict initial dry biomass of each of our individuals using measurements of the same morphological characters taken at the beginning of the experiment prior to their transplant in the field. The range of initial plant sizes predicted was included within the range of final plant sizes, indicating that our predictions do not represent extrapolations from the data used to parameterize the model. Details of the final predictive model are presented below.

#### Final model

Initial.dry.biomass ~ Height + Rhizome.length + Rhizome.node.thickness + Number.of.leaves

**Table:**

Coefficients	Estimate	Standard error
(Intercept)	−1.54E−02***	2.63E−03
Height	2.90E−04***	7.92E−05
Rhizome.length	8.99E−05***	7.45E−06
Rhizome.node.thickness	9.36E−03***	7.46E−04
Number.of.leaves	5.29E−03***	4.59E−04

Significance is indicated as the following: **p* < .05, ***p* < .01, ****p* < .001.

## APPENDIX 2

### Post hoc pairwise comparisons of final survival and dry biomass among elevations of origin by elevation of transplant

Post hoc pairwise comparisons among elevations of origin at each elevation of transplant were performed respectively for survival (A) and biomass (B) in order to test for local adaptation, which would be observed as a better performance of a population when transplanted to its elevation of origin versus foreigners transplanted at this same elevation. Results were averaged over transects and *p*‐values were adjusted using the Tukey method for comparing a family of three estimates using R package lsmeans (Lenth, 2016). Significance is indicated as the following: **p* < .05, ***p* < .01, ****p* < .001.

**Table:**

(A) Survival					
Contrast among elevations of origin	Estimate	*SE*	*df*	*z* Ratio	*p* Value
Elevation of transplant = Low					
Low‐Mid	−0.4925	0.5766	NA	−0.8542	.6692
Low‐High	−0.5127	0.5598	NA	−0.9158	.6303
Mid‐High	−0.0202	0.5610	NA	−0.0360	.9993
Elevation of transplant = Mid					
Low‐Mid	−0.2294	0.7064	NA	−0.3248	.9435
Low‐High	0.6521	0.6168	NA	1.0574	.5406
Mid‐High	0.8816	0.6520	NA	1.3520	.3664
Elevation of transplant = High					
Low‐Mid	−0.2108	0.5098	NA	−0.4135	.9101
Low‐High	−0.4603	0.5197	NA	−0.8857	.6493
Mid‐High	−0.2495	0.5065	NA	−0.4926	.8749

(B) Biomass					
Contrast among elevations of origin	Estimate	*SE*	*df*	*t* Ratio	*p* Value
Elevation of transplant = Low					
Low‐Mid	0.0268	0.0198	295.98	1.354	.3666
Low‐High	0.0386	0.0192	295.09	2.005	.1127
Mid‐High	0.0118	0.0179	291.47	0.656	.7889
Elevation of transplant = Mid					
Low‐Mid	0.0520	0.0146	287.83	3.574	.0012*
Low‐High	0.0380	0.0153	290.52	2.492	.0353*
Mid‐High	−0.0140	0.0153	293.12	−0.919	.6284
Elevation of transplant = High					
Low‐Mid	−0.0194	0.0178	295.41	−1.092	.5199
Low‐High	−0.0205	0.0176	299.5	−1.166	.4744
Mid‐High	−0.0010	0.0166	295.43	−0.062	.9979

## References

[ece33947-bib-0001] Ackerly, D. D. (2003). Community assembly, niche conservatism, and adaptive evolution in changing environments. International Journal of Plant Sciences, 164, S165–S184. https://doi.org/10.1086/368401

[ece33947-bib-0002] Albert, C. H. , Grassein, F. , Schurr, F. M. , Vieilledent, G. , & Violle, C. (2011). When and how should intraspecific variability be considered in trait‐based plant ecology? Perspectives in Plant Ecology, Evolution and Systematics, 13, 217–225. https://doi.org/10.1016/j.ppees.2011.04.003

[ece33947-bib-0003] Angert, A. L. , & Schemske, D. W. (2005). The evolution of species’ distributions: Reciprocal transplants across the elevation ranges of *Mimulus cardinalis* and *M. lewisii* . Evolution, 59, 1671–1684. https://doi.org/10.1111/j.0014-3820.2005.tb01817.x 16329239

[ece33947-bib-0004] Bates, D. , Maechler, M. , Bolker, B. , & Walker, S. (2015). Fitting linear mixed‐effects models using lme4. Journal of Statistical Software, 67, 1–48.

[ece33947-bib-0005] Berg, H. (2000). Differential seed dispersal in *Oxalis acetosella*, a cleistogamous perennial herb. Acta Oecologica, 21, 109–118. https://doi.org/10.1016/S1146-609X(00)00118-1

[ece33947-bib-0006] Brown, C. D. , & Vellend, M. (2014). Non‐climatic constraints on upper elevational plant range expansion under climate change. Proceedings of the Royal Society B: Biological Sciences, 281, 20141779 https://doi.org/10.1098/rspb.2014.1779 25253462 10.1098/rspb.2014.1779 PMC4211457

[ece33947-bib-0007] Chapin, F. S. , & Chapin, M. C. (1981). Ecotypic differentiation of growth processes in *Carex aquatilis* along latitudinal and local gradients. Ecology, 62, 1000–1009.

[ece33947-bib-0008] Chevin, L.‐M. , Collins, S. , & Lefèvre, F. (2013). Phenotypic plasticity and evolutionary demographic responses to climate change: Taking theory out to the field. Functional Ecology, 27, 967–979. https://doi.org/10.1111/j.1365-2435.2012.02043.x

[ece33947-bib-0009] Clausen, J. , Keck, D. D. , & Hiesey, W. M. (1940). Experimental studies on the nature of species I. Effect of varied environments on Western North American plants. Washington, DC: Carnegie Institution of Washington.

[ece33947-bib-0010] Cornwell, W. K. , & Ackerly, D. D. (2009). Community assembly and shifts in plant trait distributions across an anvironmental gradient in coastal California. Ecological Monographs, 79, 109–126. https://doi.org/10.1890/07-1134.1

[ece33947-bib-0011] Davis, M. B. , Shaw, R. G. , & Etterson, J. R. (2005). Evolutionary responses to changing climate. Ecology, 86, 1704–1714. https://doi.org/10.1890/03-0788

[ece33947-bib-0012] Emery, R. J. M. , Chinnappa, C. C. , & Chmielewski, J. G. (1994). Specialization, plant strategies and phenotypic plasticity in populations of *Stellaria longipe* along an elevational gradient. International Journal of Plant Sciences, 155, 203–219. https://doi.org/10.1086/297160

[ece33947-bib-0013] Etterson, J. R. (2004). Evolutionary potential of *Chamaecrista fasciculata* in relation to climate change. I. Clinal patterns of selection along an environmental gradient in the Great Plains. Evolution, 58, 1446–1458. https://doi.org/10.1111/j.0014-3820.2004.tb01726.x 15341148 10.1111/j.0014-3820.2004.tb01726.x

[ece33947-bib-0014] Etterson, J. R. , & Shaw, R. G. (2001). Constraint to adaptive evolution in response to global warming. Science, 294, 151–154. https://doi.org/10.1126/science.1063656 11588260 10.1126/science.1063656

[ece33947-bib-0015] Franks, S. J. , Sim, S. , & Weis, A. E. (2007). Rapid evolution of flowering time by an annual plant in response to a climate fluctuation. Proceedings of the National Academy of Sciences of the United States of America, 104, 1278–1282. https://doi.org/10.1073/pnas.0608379104 17220273 10.1073/pnas.0608379104 PMC1783115

[ece33947-bib-0016] Franks, S. J. , Weber, J. J. , & Aitken, S. N. (2014). Evolutionary and plastic responses to climate change in terrestrial plant populations. Evolutionary Applications, 7, 123–139. https://doi.org/10.1111/eva.12112 24454552 10.1111/eva.12112 PMC3894902

[ece33947-bib-0017] Frei, E. R. , Ghazoul, J. , Matter, P. , Heggli, M. , & Pluess, A. R. (2014). Plant population differentiation and climate change: Responses of grassland species along an elevational gradient. Global Change Biology, 20, 441–455. https://doi.org/10.1111/gcb.12403 24115364 10.1111/gcb.12403

[ece33947-bib-0018] Garzon, M. B. , Alia, R. , Robson, T. M. , & Zavala, M. A. (2011). Intra‐specific variability and plasticity influence potential tree species distributions under climate change. Global Ecology and Biogeography, 20, 766–778. https://doi.org/10.1111/j.1466-8238.2010.00646.x

[ece33947-bib-0019] Geber, M. A. , & Griffen, L. R. (2003). Inheritance and natural selection on functional traits. International Journal of Plant Sciences, 164, s21–s42. https://doi.org/10.1086/368233

[ece33947-bib-0020] Gienapp, P. , Teplitsky, C. , Alho, J. , Mills, J. , & Merilä, J. (2008). Climate change and evolution: Disentangling environmental and genetic responses. Molecular Ecology, 17, 167–178. https://doi.org/10.1111/j.1365-294X.2007.03413.x 18173499 10.1111/j.1365-294X.2007.03413.x

[ece33947-bib-0021] Givnish, T. J. (1982). On the adaptive significance of leaf height in forest herbs. The American Naturalist, 120, 353–381. https://doi.org/10.1086/283995

[ece33947-bib-0022] Gonzalo‐Turpin, H. , & Hazard, L. (2009). Local adaptation occurs along altitudinal gradient despite the existence of gene flow in the alpine plant species *Festuca eskia* . Journal of Ecology, 97, 742–751. https://doi.org/10.1111/j.1365-2745.2009.01509.x

[ece33947-bib-0023] Hautier, Y. , Randin, C. F. , Stocklin, J. , & Guisan, A. (2009). Changes in reproductive investment with altitude in an alpine plant. Journal of Plant Ecology, 2, 125–134. https://doi.org/10.1093/jpe/rtp011

[ece33947-bib-0024] Jump, A. S. , & Penuelas, J. (2005). Running to stand still: Adaptation and the response of plants to rapid climate change. Ecology Letters, 8, 1010–1020. https://doi.org/10.1111/j.1461-0248.2005.00796.x 10.1111/j.1461-0248.2005.00796.x 34517682

[ece33947-bib-0025] Jung, V. , Albert, C. H. , Violle, C. , Kunstler, G. , Loucougaray, G. , & Spiegelberger, T. (2014). Intraspecific trait variability mediates the response of subalpine grassland communities to extreme drought events. Journal of Ecology, 102, 45–53. https://doi.org/10.1111/1365-2745.12177

[ece33947-bib-0026] Kawecki, T. J. , & Ebert, D. (2004). Conceptual issues in local adaptation. Ecology Letters, 7, 1225–1241. https://doi.org/10.1111/j.1461-0248.2004.00684.x

[ece33947-bib-0027] Lajoie, G. , & Vellend, M. (2015). Understanding context dependence in the contribution of intraspecific variation to community trait–environment matching. Ecology, 96, 2912–2922. https://doi.org/10.1890/15-0156.1 27070011 10.1890/15-0156.1

[ece33947-bib-0028] Lande, R. , & Arnold, S. J. (1983). The measurement of selection on correlated characters. Evolution, 37, 1210–1226. https://doi.org/10.1111/j.1558-5646.1983.tb00236.x 28556011 10.1111/j.1558-5646.1983.tb00236.x

[ece33947-bib-0029] Laughlin, D. C. , & Messier, J. (2015). Fitness of multidimensional phenotypes in dynamic adaptive landscapes. Trends in Ecology and Evolution, 30, 487–496. https://doi.org/10.1016/j.tree.2015.06.003 26122484 10.1016/j.tree.2015.06.003

[ece33947-bib-0030] Leimu, R. , & Fischer, M. (2008). A meta‐analysis of local adaptation in plants. PLoS ONE, 3, 1–8. 10.1371/journal.pone.0004010 PMC2602971 19104660

[ece33947-bib-0031] Lenth, R. V. (2016). Least‐squares means: The R package lsmeans. Journal of Statistical Software, 69, 1–33.

[ece33947-bib-0032] Lepš, J. , de Bello, F. , Šmilauer, P. , & Doležal, J. (2011). Community trait response to environment: Disentangling species turnover vs intraspecific trait variability effects. Ecography, 34, 856–863.

[ece33947-bib-0033] Meziane, D. , & Shipley, B. (1999). Interacting determinants of specific leaf area in 22 herbaceous species: Effects of irradiance and nutrient availability. Plant, Cell & Environment, 22, 447–459. https://doi.org/10.1046/j.1365-3040.1999.00423.x

[ece33947-bib-0034] Mitchell, R. M. , & Bakker, J. D. (2014). Intraspecific trait variation driven by plasticity and ontogeny in *Hypochaeris radicata* . PLoS ONE, 9, e109870 https://doi.org/10.1371/journal.pone.0109870 25333738 10.1371/journal.pone.0109870 PMC4204820

[ece33947-bib-0035] Muscarella, R. , & Uriarte, M. (2016). Do community‐weighted mean functional traits reflect optimal strategies? Proceedings of the Royal Society B, 283, 20152434 https://doi.org/10.1098/rspb.2015.2434 27030412 10.1098/rspb.2015.2434 PMC4822452

[ece33947-bib-0036] Nicotra, A. B. , Atkin, O. K. , Bonser, S. P. , Davidson, A. M. , Finnegan, E. J. , Mathesius, U. , … van Kleunen, M. (2010). Plant phenotypic plasticity in a changing climate. Trends in Plant Science, 15, 684–692. https://doi.org/10.1016/j.tplants.2010.09.008 20970368 10.1016/j.tplants.2010.09.008

[ece33947-bib-0037] Ouranos (2015). Vers l'adaptation. Synthèse des connaissances sur les changements climatiques au Québec (2015 ed., p. 415). Montréal, QC: Ouranos.

[ece33947-bib-0038] Packham, J. , & Willis, A. (1977). The effects of shading on *Oxalis acetosella* . The Journal of Ecology, 65, 619–642. https://doi.org/10.2307/2259505

[ece33947-bib-0039] Parmesan, C. (2006). Ecological and evolutionary responses to recent climate change. Annual Review of Ecology, Evolution, and Systematics, 37, 637–669. https://doi.org/10.1146/annurev.ecolsys.37.091305.110100

[ece33947-bib-0040] Poll, M. , Naylor, B. J. , Alexander, J. M. , Edwards, P. J. , & Dietz, H. (2009). Seedling establishment of Asteraceae forbs along altitudinal gradients: A comparison of transplant experiments in the native and introduced ranges. Diversity and Distributions, 15, 254–265. https://doi.org/10.1111/j.1472-4642.2008.00540.x

[ece33947-bib-0041] Price, E. A. C. , & Marshall, C. (1999). Clonal plants and environmental heterogeneity – An introduction to the proceedings. Plant Ecology, 141, 3–7. https://doi.org/10.1023/A:1009838300691

[ece33947-bib-0042] R Core Team (2015). R: A language and environment for statistical computing. Vienna, Austria: R Foundation for Statistical Computing.

[ece33947-bib-0043] Rasband, W. S. (1997–2012). ImageJ v. 1.45s. Bethesda, MD: National Institutes of Health.

[ece33947-bib-0044] Reed, T. E. , Schindler, D. E. , & Waples, R. S. (2011). Interacting effects of phenotypic plasticity and evolution on population persistence in a changing climate. Conservation Biology, 25, 56–63. https://doi.org/10.1111/j.1523-1739.2010.01552.x 20646016 10.1111/j.1523-1739.2010.01552.x PMC3084585

[ece33947-bib-0045] Reich, P. B. , Walters, M. B. , Ellsworth, D. S. , & Uhl, C. (1994). Photosynthesis‐nitrogen relations in Amazonian tree species. I. Patterns among species and communities. Oecologia, 97, 62–72. https://doi.org/10.1007/BF00317909 28313590 10.1007/BF00317909

[ece33947-bib-0046] Richardson, B. A. , Chaney, L. , Shaw, N. L. , & Still, S. M. (2017). Will phenotypic plasticity affecting flowering phenology keep pace with climate change? Global Change Biology, 23, 2499–2508. https://doi.org/10.1111/gcb.13532 27739159 10.1111/gcb.13532

[ece33947-bib-0047] Savage, J. , & Vellend, M. (2015). Elevational shifts, biotic homogenization and time lags in vegetation change during 40 years of climate warming. Ecography, 38, 546–555. https://doi.org/10.1111/ecog.01131

[ece33947-bib-0048] Shipley, B. , de Bello, F. , Cornelissen, J. H. , Laliberté, D. C. , & Reich, P. B. (2016). Reinforcing loose foundation stones in trait‐based plant ecology. Oecologia, 180, 923–931. https://doi.org/10.1007/s00442-016-3549-x 26796410 10.1007/s00442-016-3549-x

[ece33947-bib-0049] Shipley, B. , Vile, D. , & Garnier, É. (2006). From plant traits to plant communities: A statistical mechanistic approach to biodiversity. Science, 314, 812–814. https://doi.org/10.1126/science.1131344 17023613 10.1126/science.1131344

[ece33947-bib-0050] Siefert, A. , Violle, C. , Chalmandrier, L. , Albert, C. H. , Taudiere, A. , Fajardo, A. , … Wardle, D. A. (2015). A global meta‐analysis of the relative extent of intraspecific trait variation in plant communities. Ecology Letters, 18, 1406–19. https://doi.org/10.1111/ele.12508 26415616 10.1111/ele.12508

[ece33947-bib-0051] Valladares, F. , Matesanz, S. , Guilhaumon, F. , Araujo, M. B. , Balaguer, L. , Benito‐Garzon, M. , … Zavala, M. A. (2014). The effects of phenotypic plasticity and local adaptation on forecasts of species range shifts under climate change. Ecology Letters, 17, 1351–1364. https://doi.org/10.1111/ele.12348 25205436 10.1111/ele.12348

[ece33947-bib-0052] Van Tienderen, P. H. , & Van Der Toorn, J. (1991). Genetic differentiation between populations of Plantago lanceolata. II. Phenotypic selection in a transplant experiment in three contrasting habitats. Journal of Ecology, 79, 43–59. https://doi.org/10.2307/2260783

